# The League of Mercy

**Published:** 1902-11-22

**Authors:** 


### THE LEAGUE OF MERCY.

The League of Mercy, in association with King Edward's Hospital Fund, is organising its winter arrangements. Its objects are to secure the co-operation of all those residents in London and the districts around who realise the privilege of rendering a measure of personal service to the sick by devoting some three days in each year to the work of the League. The treasurer, Sir Henry Burdett, will be glad to receive offers of co-operation from London residents addressed to him at the offices of the League, 29 Southampton Street, Strand, W.C. Sir Henry Burdett, on the invitation of the presidents of some of the metropolitan and home counties divisions of the League of Mercy, has consented to show the actual work done in the hospitals of the metropolis and at the seat of war by a series of living pictures. The subjects included are the following:—With the Out-patients; With the In-patients; Life in the Wards; Visiting Day; Before the Days of Chloroform; The Operation Theatre; The Convalescent Home; Sisters and Nurses; Hospitals and the War.

A most successful meeting was held at Colchester on Tuesday evening, when Sir Weetman Pearson, Bart., M.P., presided. At the close of the exhibition of pictures Sir Henry Burdett delivered an eloquent and powerful address on behalf of the London hospitals. The following further fixtures have been made:—On Thursday, November 27th, at the Grand Hall, Bromley, at 8 p.m. (president, Mr. E. A. Hambro); on Friday, November 28th, at the Pump Room, Tunbridge Wells, at 8 p.m. (president, The Marquess Camden); on Friday, December 12th, at the Town Hall, St. Pancras, at 8 p.m. (president, Mr. T. H. Idris, L.C.C.).

